# Real-time synthesis and detection of plasmonic metal (Au, Ag) nanoparticles under monochromatic X-ray nano-tomography

**DOI:** 10.1038/s41598-020-77853-x

**Published:** 2020-11-30

**Authors:** Amardeep Bharti, Keun Hwa Chae, Navdeep Goyal

**Affiliations:** 1 Department of Physics, Panjab University, Chandigarh, 160014 India; 2 Inter-University Accelerator Centre, New Delhi, 110067 India; 3 Pohang Accelerator Laboratory, Gyeongbuk, 37673 South Korea; 4 Advanced Analysis Center, Korea Institute of Science and Technology, Seoul, 02792 South Korea

**Keywords:** Chemical engineering, Energy, Green chemistry, Materials chemistry, Photochemistry, Chemical synthesis, Photovoltaics, Chemical engineering, Electronic devices, Photocatalysis, Solar cells, Nanophotonics and plasmonics, Photonic crystals, Metals and alloys, Nanoparticles, Characterization and analytical techniques, Design, synthesis and processing, Imaging techniques, Spectrophotometry, Nanoparticles, Nanophotonics and plasmonics, Imaging and sensing, Spectrophotometry, Green photonics, Imaging techniques, Chemical physics

## Abstract

Plasmonic nanostructures are of immense interest of research due to its widespread applications in microelectronics, photonics, and biotechnology, because of its size and shape-dependent localized surface plasmon resonance response. The great efforts have been constructed by physicists, chemists, and material scientists to deliver optimized reaction protocol to tailor the size and shape of nanostructures. Real-time characterization emerges out as a versatile tool in perspective to the optimization of synthesis parameters. Moreover, in the past decades, radiation-induced reduction of metallic-salt to nanoparticles dominates over the conventional direct chemical reduction process which overcomes the production of secondary products and yields ultra-high quality and pure nanostructures. Here we show, the real-time/in-situ synthesis and detection of plasmonic (Au andAg) nanoparticles using single synchrotron monochromatic 6.7 keV X-rays based Nano-Tomography beamline. The real-time X-ray nano-tomography of plasmonic nanostructures has been first-time successfully achieved at such a low-energy that would be leading to the possibility of these experiments at laboratory-based sources. In-situ optical imaging confirms the radiolysis of water molecule resulting in the production of $e_{aq}^-,\,OH^\bullet ,$ and $O_2^-$ under X-ray irradiation. The obtained particle-size and size-distribution by X-ray tomography are in good agreement to TEM results. The effect of different chemical environment media on the particle-size has also been studied. This work provides the protocol to precisely control the size of nanostructures and to synthesize the ultrahigh-purity grade monodisperse nanoparticles that would definitely enhance the phase-contrast in cancer bio-imaging and plasmonic photovoltaic application.

## Introduction

Plasmonic nanostructures are the promising tools for widespread application especially for phase contrast in bioimaging^1–3^ and light trapping in the solar cell/photodetectors due to their conspicuous size and shape dependent localized surface plasmon resonance response^4,5^. Plasmonic nanoparticles not only produces the phase-contrast in image detection^6^ but simultaneously cure the cancer tissue^7^ because of its antibacterial properties^8^. On the other side, Plasmonic nanostructures are widely used in the photovoltaic cells due to its topology and morphology dependent unique optical, charge storage, absorption coefficient, good energy band-gap, and light trapping properties^9–11^. The unique Surface Plasmon Resonance Response of these nanostructures lies in the visible to IR-region which makes them suitable for the energetic solar photons trapping and resulting in enhancement in efficiency of the photovoltaic cells^4,9^. The properties of these polaritons could be tuned by optimizing the size and shape of nanostructures^10,11^. In order to optimize the specific response of the material, in-situ/real-time characterizations emerge as a vital tool to probe the materials properties^12,13^. Another aspect of in-situ characterization is to avoid the material wastage and time. From the industrial application point of view, the material should be of high purity grade without any secondary product contamination along with the sustainable properties. As far as the chemical synthesis roots are concerned, various parameters have been optimized for the desired characteristics materials^14,15^. In this regards, real-time characterization provide the feasibility to monitor the materials characteristics to optimize the desired properties.

In our previous work, the size and shape dependent properties of the plasmonic nanostructure fabricated under the irradiation of low and high-frequency radiations has been studied^16,17^. The results clearly depicted the dominant role of radiation-induced synthesis over the direct chemical reduction process along with providing the feasibility of in-situ characterization. The radiation induced synthesis protocol prevents the formation of unbidden secondary products because of the absence of external chemical reducing agents.

Moreover, Material Science is a 3D-science, in which the reconstruction of microstructures incorporating anisotropic grains, intergranular phases, island formation on surfaces, crack distributions and deformed structures, etc., led to the interesting physics at the bottom^18^. In the last two decades, such material modification has been successfully achieved with the improving ion-beam driven instrumentations. So the characterization techniques which would enable researchers to investigate the 3D structures rather than a projection, are of interest in material science^18–20^.

In the microstructure imaging, optical coherence tomography based on the coherent properties of light to fetch structural feature of heterogeneous optically opaque samples is widely used with the advancement of its resolution ~ 2 μm and imaging speed^19^. The detection of metal oxides in the tissues has been successfully achieved using optical coherence tomography^21,22^.

Metals (Au and Ag) have a strong X-ray attenuation coefficient among other elements naturally present in humans. Therefore, the accretion of Au/Ag NP in tumors would significantly enhance the X-ray attenuation, resulting in high contrast between tumor and healthy tissues on tomography images^23^. At present deoxyglucose-labeled AuNP are widely accepted as potential X-ray tomography contrast agent^24^. The 10-fold enhancement in tumor detection on X-ray tomography has been observed after the incorporation of 2.6 nm AuNP^25^. Moreover, M-NP not only enhance the image contrast also serve in treatment. The significant enhancement in radiation therapy has been observed in the presence of 1.9 nm AuNP^26^. The Auger de-excitation process in Ag-NP under gamma radiation leading to effective DNA breaking in a short distance less than the size of a single cell resulting in damage to tumor cell without harming other body-tissues^23^. This short-range therapeutic effect makes AuNP a potential element for selective tumor cells detection and treatment under photon-based radiation therapy.

In case of plasmonics driven photovoltaic devices, the metallic nanoparticles/nanostructures have been employed for light trapping via interaction with surface polaritons. Such plasmonic nanostructures have been incorporated at the surface, back contact and in-bulk of the active layer of the device^9^. So it is important to consider the 3D-diffusion of particles to understand the dopant profile. In the past decades, a lot of efforts have been imparted focusing on this particular issue. The 3D doping profile of nanostructures have been studied by atom probe tomography which would be of interest in the plasmonic photovoltaic cell^20^. Another way of 3D imaging is by using discrete tomography in which 2D projection images obtained from different angles by electron microscopy can be reconstructed for the 3D tomography by applying the discrete algebraic reconstruction technique (DART)^27^.

The availability of highly collimated and intense X-ray beams from the synchrotron source has begun the evolution of X-ray based characterization techniques with its widespread applications in physical, life and biological sciences^28^. Moreover, it allows the feasibility to use multiple characterization techniques simultaneously.

In this work, we successfully achieve the task of fabrication of in-situ plasmonic nanostructure by the reduction of metal ions $(M^+)$ to the zero-valent $(M^0)$ nanoparticles in a liquid phase using water radiolysis along with the online characterization by X-ray nano-tomography/imaging technique under synchrotron monochromatic radiations. In these experiments the same monochromatic hard-X-ray beam has been utilized for the synthesis/fabrication and in-situ detection of plasmonic nanostructures which will definitely enlighten the concepts behind the anomalous behavior of nanomaterials. The successful outcome of this work will lead the feasibility of synthesizing the metal-polymer conjugates and in-situ probing the charge transport properties for bio-imaging and solar cell applications.

## X-ray nanotomography

### Experimental beamline setup and evaluation procedure

In material science, particle size and shape is an important factor to optimize their characteristics. Therefore, material imaging is a prime choice and can be executing with numerous probes, i.e., light, electrons, neutrons, ultrasound and X-rays^18^. X-rays allows the feasibility for in-situ experimentation to widespread range from thin to thick samples due to the high transmission and non-destructive property^29,30^. Although, low absorption affects the image contrast, but can be resolved by phase-contrast technique. It utilize the phase-difference between 0*th* and *nth*-order spatial frequencies. The contrast can be explained in terms of wave-optical approach as ray-refraction by the object. The small deviation in the direction ~ 10 μrad is sufficient to produce noticeable variation in intensity, after propagation over the ~ 1 m^31^.

In wave-optical approach, the object is characterized by its complex transmission function *T* as^32^:

1 $$\begin{aligned} u(x,y) = T(x,y) u_0(x,y) \end{aligned}$$

where *u* and $u_0$ defines the monochromatic field mode (downstream and upstream respectively) of the object at (*x*, *y*) point of the object plane. The complex transmission function *T* includes the real and imaginary part of refractive index of material and can be defined as

2 $$\begin{aligned} T(x,y) = A(x,y) e^{i\phi (x,y)} \end{aligned}$$

where *A* and $\phi$ are the absorption and phase modulation respectively.

3 $$\begin{aligned} A(x,y)= & {} exp \Big (-\frac{1}{2} \int \mu (x,y,z)dz \Big ) \end{aligned}$$

4 $$\begin{aligned} \phi (x,y)= & {} \frac{2\pi }{\lambda } \int (n(x,y,z) - 1)dz \end{aligned}$$

where $\mu$ and *n* are the absorption coefficients and real part of refractive index, respectively. The integral term over the *z* is due to the propagation direction in the object. In case of X-rays, the refractive index *n* of elements depends on their electron density as^31^

5 $$\begin{aligned} n = 1 - (N_0/A_m)\rho _mr_0(Z + f'_{disp})\lambda ^2 \end{aligned}$$

where $N_0$ is the Avogardro’s number, $A_m=$ atomic mass, $Z=$ atomic number, $\rho _m =$ material density, $r_0 =$ classical electron radius, $f'_{disp} =$ real part of dispersion correction and $\lambda =$ wavelength. Due to high mobility and electron density, metals are of first choice in the imaging techniques. Moreover, In case of the metallic nanoparticles, surface electron density enhances and produce phase modulation/contrast. That is why, metallic nanoparticles, especially AuNP being antibacterial and non-toxic is widely used for image-contrast in biomedical applications.

**Figure 1:**
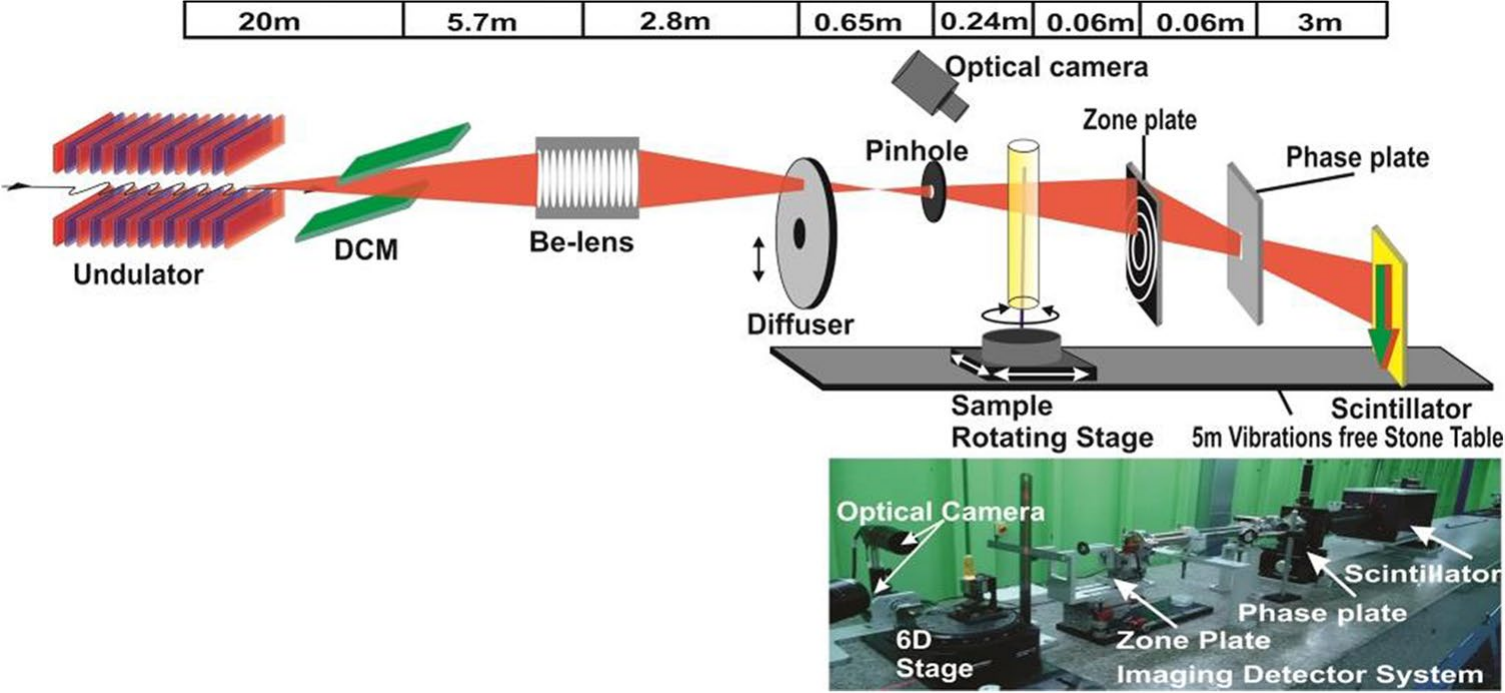
Optical layout of 7C-X-ray imaging beamline showing its major components alongside the photographs showing detailed beamline setup, inset shows the illustration of radiation induced water-radiolysis leading to reduction/oxidation of metallic-salts.

The optical layout and image showing the relative distance of the major components of 7C-XNI hard X-ray nanotomography beamline at Pohang light source are shown in the Fig. 1. The beamline emerges from the 1.4 m hybrid undulator having a period of 20 mm to produce high flux X-rays of power 2.95 kW at 3.0 GeV storage beam energy and 400 mA current. The Silicon (Si-111) double crystal monochromator (DCM-Vactron, Korea) has been used to tune and monochromatize $(\Delta E/E \sim 10^{-4})$ the X-rays beam. The DCM is allowed to work under liquid nitrogen cooling system and a thick diamond window 200 μm of 1 cm diameter is placed in front of the DCM to reduce the thermal effects generated by high flux X-rays^33^.

The series of 10 parabolic beryllium crystal (RXOPTICS, Germany) lenses of each 1 mm in diameter having an effective aperture of $\sim 0.6\,mm$ (because of the reduction due to absorption) is used to collimate the X-rays beam^34^. Each lens offers the focal length of 3.25 m at 6.7 keV beam energy. The photon flux at the focus point is estimated to be in the order of $\sim 10^{11} \,photons\,s^{-1}$. To ensure the reduction of spatial coherency and homogenous illumination a diffuser and a pinhole of 40 μm has been installed before the experimental stage. The sample is placed on the piezo-driven three-axis scanning stage (AG-LS25-Newport) and rotating (ABRS-150MP-Areotech) 6D stage. The nanotomography has been carried out using set of Zone plate, a phase plate, and scintillator detector. The objective zone plate is made up of 1.0 μm thick and 140 μm diameter tungsten (Zoneplates, UK) plate having $\sim 30\%$ efficiency. The first order focal length comes out to be $40\,mm$ at beam energy of 6.7 keV. In order to set the contrast of imaging, a $3.78 \,\pm <0.04 \,\mu m$ thick aluminum phase plate having a $10 \,\mu m$ hole is inserted at the focal point of zone plane, which offers the $\pi /2$ phase shift in the diffracted beam to produce a darker image in the bright field.

The detector system contains an $18\,\mu m$ thin, $10\,mm$ diameter *Tb* : *LSO* scintillator crystal (FEE, Germany) and X20 optical microscope. In order to eliminate the blur effect, all optical components of beamline are operated under a vacuum of $10^{-3}$ torr and placed on the 5 m long and 30cm thick vibration-free granite plate^34^.

## Target material processing

All the chemicals of high purity $(99.999\%)$ grade $AgNO_3$, $AuCl_3$, and *PVP* were purchased from Sigma Aldrich and used as it is without any further purification. The precursor solution was formed by dissolving the metal-salt and capping agent (PVP) in high purity HPLC reagent water (SAMCHUN chemicals-Korea).

**Figure 2:**
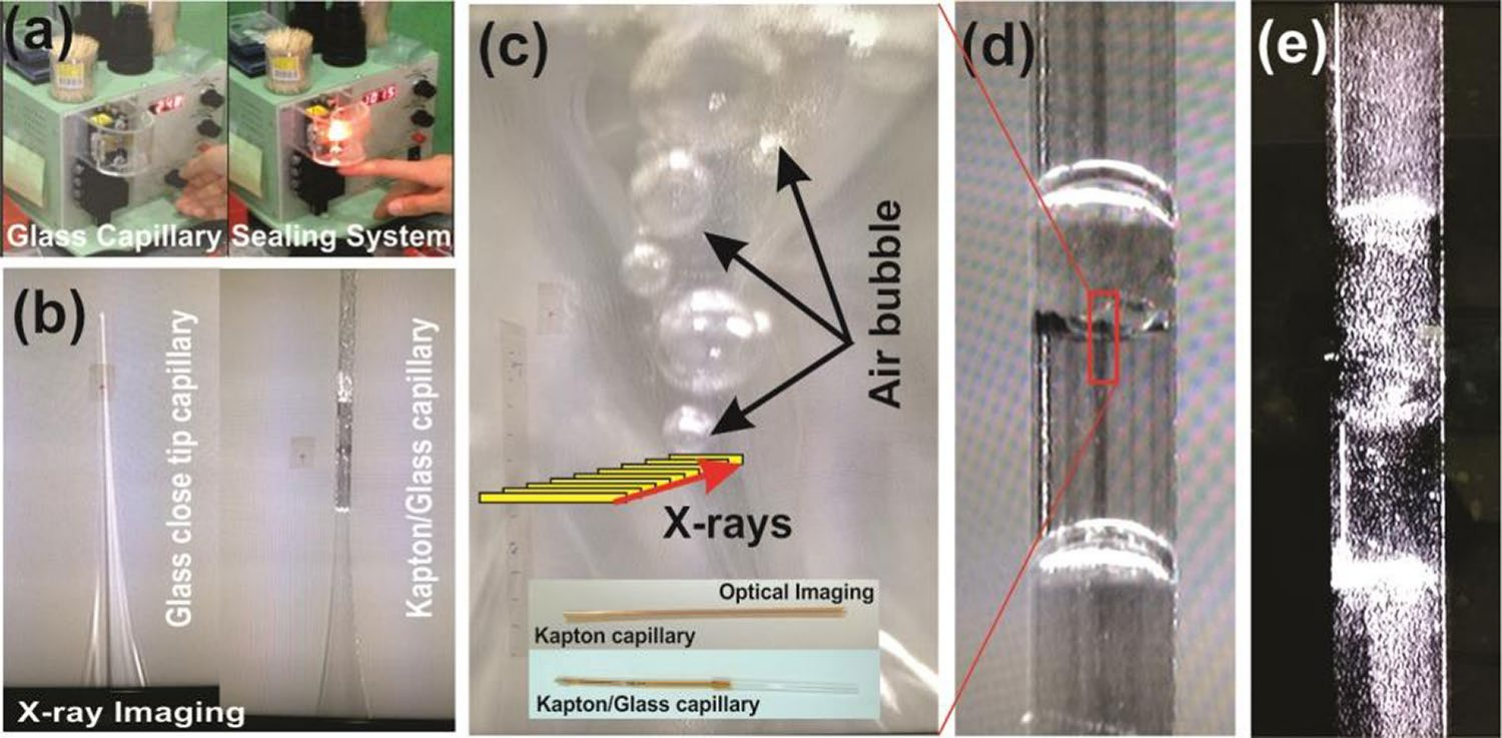
Glass capillary sealing system (**a**) showing: X-ray imaging of Kapton/glass capillary filled with precursor solution (**b**), Optical imaging showing the production of air bubbles during X-ray irradiation (**c**), images of Kapton capillary at beginning (**d**) and after the exposure (**e**) of 6.7 keV monochromatic X-rays under laser-light exposure.

The precursor solution was filled in the Kapton-Polyimide tube (Cole-Parmer(Illinois)) having an outer diameter of $0.0615'' \pm 0.0005''$ and an inner diameter $0.0575'' \pm 0.0005''$ with the wall thickness of $0.00200'' \pm 0.00025''$. To close the one end of Kapton tube, a closed end tip glass capillary has been injected. A dedicated glass capillary sealing system has been used to close the end of a capillary as shown in the Fig. 2.

## Results and discussion

Real-time/in-situ detection of *Au* nanopaticles during irradiation of precursor samples under same beamline has been investigated using undulator based X-ray tomography beamline (7C-XNI) at pohang accelerator laboratory (PAL-South korea). The X-ray image of target sample at before starting the experiment (0 irradiation time) has been recorded as shown in Fig. 2b. Radiations induced material synthesis has been widely accepted for the production of high purity grade nanomaterials because of its precise control over the reaction rate by means of dosimetry^35,36^. In order to investigate the fundamental process of radiation induced reduction of metallic-salt, the monochromatic X-rays of energy 6.7 keV much less than the $K-L$ edge-energy of *Au* has been selected for irradiation. Simultaneously, the imaging detector system has shown good resolution at 6.7 keV as determined by the previous beamline-imaging experiments^34^. There would be no external reducing agent involved in the system, therefore the chemical reaction will only be triggered under the effect of water putrefaction. At present, water decomposition using radiolysis process is carried out by ionizing radiation, i.e. radiation from the decay of radioactive materials, accelerated charged particles (electrons, protons and ions) and X-rays^37–40^. The water putrefaction is governed around the radiations-track and directly proportional to the linear energy transfer $(LET=-dE/dl)$ in the medium per unit path-length^38^. The mechanism is mainly categorised in three steps: physical stage $(1 \,fs)$, physico-chemical stage 10^−15υ^10^−12^ s and chemical stage 10^−12υ^10^−16^ s. The initial two stages involve the radiation energy deposition to decompose the water-molecule as^17^:

$$\begin{aligned} H_2O \,\,\, \overrightarrow{_{X-rays}} \,\,\, e^-_{aq} + OH^\bullet + O_2^- + H^\bullet , + H_2O_2 + H_2. \end{aligned}$$

**Figure 3:**
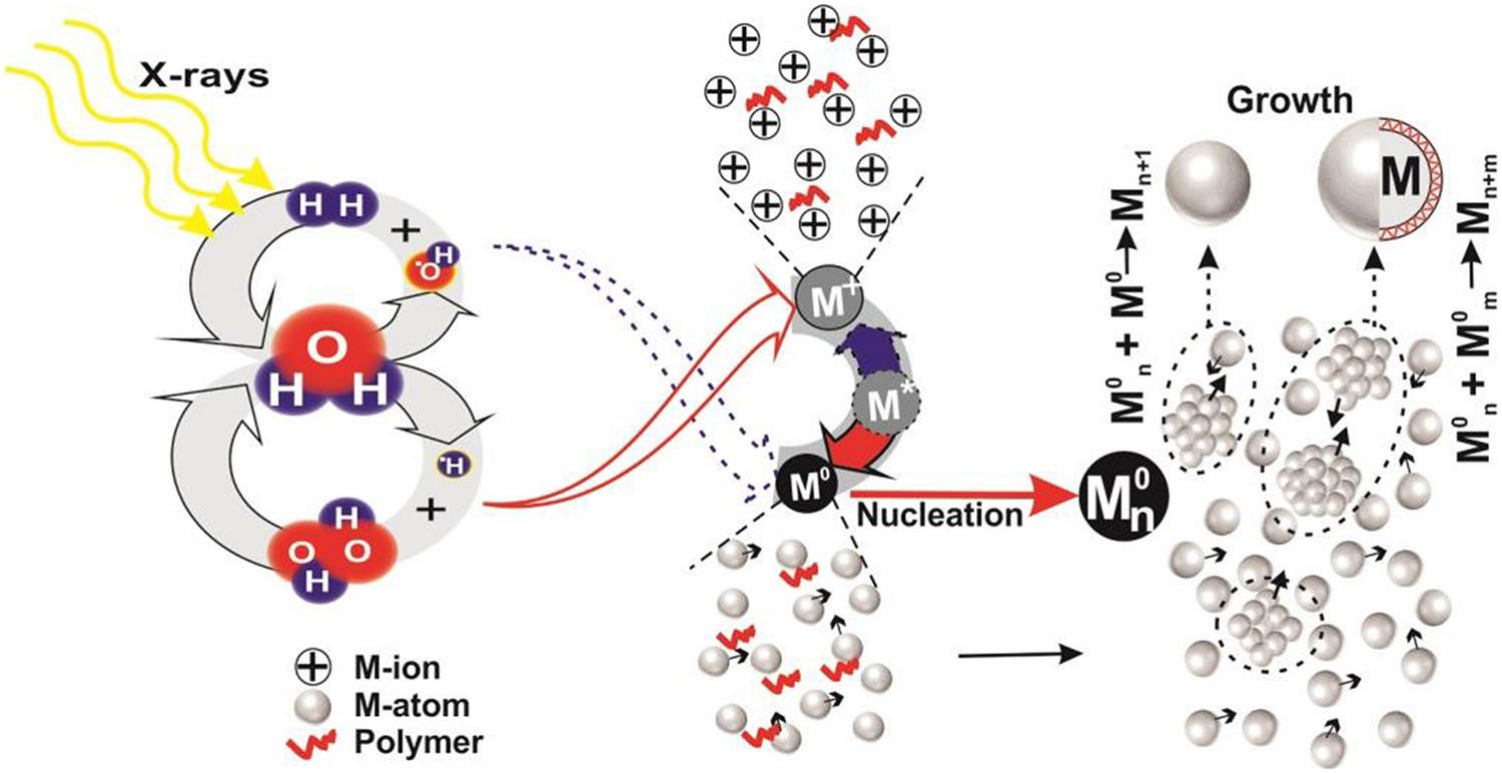
Scheme of metal-salt reduction to nanostructure and particle size growth under X-ray induced water-radiolysis.

The photon flux $F_{Ph}$ at 6.7  keV was in order of $\sim 10^9 \,ph/s$, therefore integrated beam energy-flux can be estimated as $E\times F_{Ph}=1.07\times 10^{-05} \,J/s$. If beam cross-sectional lies inside the irradiated cell parameters, the expression for integrated dose (Gy) imparted to the sample is expressed as^16^:

6 $$\begin{aligned} ID_{integrated \,dose} = \frac{E\times F_{Ph} \times \%abs}{t_{cell} \times \rho _{sample}} \times T_{irradiation \,time} \end{aligned}$$

where *t* is the irradiation-cell thickness, $\rho$ is the sample density and *T* is the irradiation time. Therefore the energy transferred to the sample is $\sim 10^{-07} \,J/s$, and the integrated dose for irradiation time of 05—60 min is about to $3.2-57.8 \,KGy$ with $10 \,Gy/s$ of dose rate. The radiolysis of water under the exposure of X-rays could be explained by simultaneous obtained optical imaging of the samples revealing the production of bubbles as shown in the Fig. 2c. The generation of bubbles on X-ray irradiation confirms the production of $O_2$ molecule, therefore the putrefaction of $H_2O$. Aqueous electrons $e^-_{aq}$ and atom radicals $H^\bullet$ are strong reducing agents towards the metal salt with standard potential $E_0(H_2O/e^-_{aq})=-2.9 \,V_{NHE}$ and $E_0(H^+/H^\bullet )=-2.3 \,V_{NHE}$ respectively, whereas hydroxyl radicals $OH^\bullet$ are strong oxidant agents with standard potential $E_0(OH^\bullet /H_2O)=+2.7 \,V_{NHE}$^37^. The putrefaction and recombination of radicals into water-molecule is continuously proceeding making an eight-loop reaction^17^ as shown in Fig. 3. This loop is mediated by the $H^\bullet$ and $OH^\bullet$ radicals maintaining the equilibrium in destruction and generation rate of molecular products as:

$$\begin{aligned}&OH^\bullet + H_2 \longrightarrow H_2O + H^\bullet \\&H^\bullet + H_2O_2 \longrightarrow H_2O + OH^\bullet \end{aligned}$$

The chemical stage involves the recombination and diffusion of the generated product in the solution which reacts with solute, resulting in the reduction of metallic-ions $(M^+)$ to zero-valent $(M^0)$ particles^17,41^.

$$\begin{aligned}&Reactants + e_{aq}^-/H^\bullet /O_2^-/OH^\bullet \longrightarrow Products,\\&M^+ + e_{aq}^-/H^\bullet /O_2^- \longrightarrow M^0,\\&M^0 + /OH^\bullet \longrightarrow M^+. \end{aligned}$$

Although $OH^\bullet$ might reverse the reaction $M^0$ to $M^+$, but the probability of such event is negligible due to the large concentration of $e_{aq}^-/H^\bullet /O_2^-$ than $OH^\bullet$^16^. Moreover the $OH^\bullet$ can be trapped using additional isopropanol or ethanol as scavengers^42^. The radiation induced reduction of metallic-salts to nano-particles mainly follow two generation scheme as shown in Fig. 3. As the zero-valent $M^0$ atoms are formed, nucleation process begins to build nanostructures. The $M^0_n$ coalesce to form the nanostructure $M^{0^n}$, which further interacts with the $M^0$ atom and synthesize the $M-NP$. If $M^{0^n}$ interact with $M^{0^m}$ nanostructure, it results in the growth $(M^{0^{n+m}})$ of $M-NP$ with larger size^16^.

The in-situ optical imaging of kapton-polyimide tube containing precursor solution at the beginning and after the exposure of 6.7  keV monochromatic X-rays is shown in the Fig. 2d,e respectively. The optical imaging of the samples was recorded under the illumination of focused laser beam on the capillary in the dark field. At the beginning of the experiment, a clear image of the kapton tube is recorded. But after the X-ray exposure, light scattering by the tube has been observed that reveals the formation of plasmonic nanoparticles. The scattering and absorption of light is an fundamental properties of plasmonic nanoparticles due to the coexistence of size and shape dependant localized surface plasmon resonance (*LSPR*) response.

**Figure 4:**
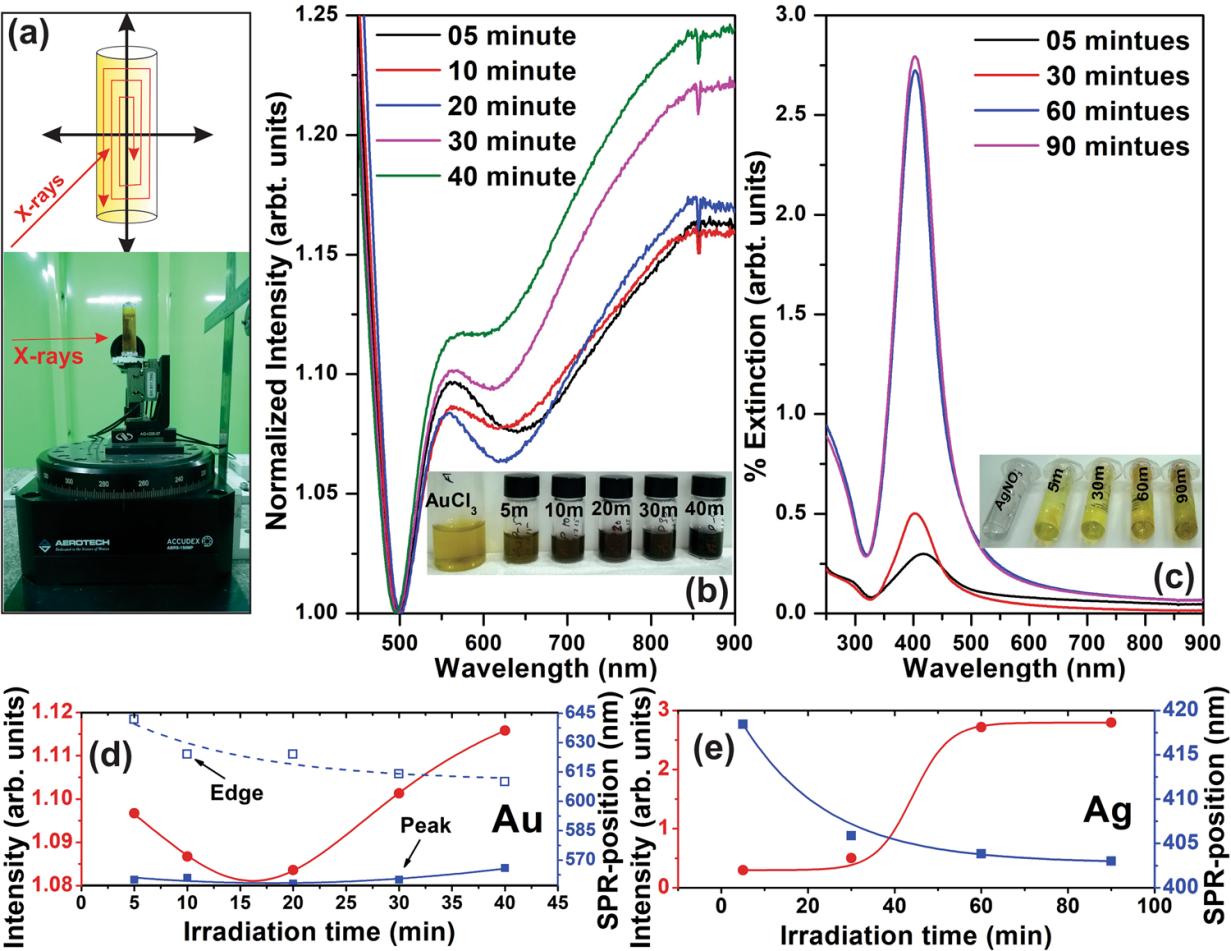
Sample target holding 6D-stage and illustration of continuous 2D motion for uniform irradiation (**a**), Extinction spectra of Au-nanostructures (**b**) and Ag-nanostructures (**c**) synthesized under the irradiation of monochromatic 6.7 keV X-rays (inset shows the color variation after the exposure) and respective extinction fitting parameter (**d**,**e**).

The effect of monochromatic X-rays of energy 6.7  keV towards the reduction of metal-salts $(AuCl_3 \,and\, AgNO_3)$ has also been investigated as a function of irradiation time. A polypropylene cell having kapton windows is specially designed for holding the precursor solution and to minimize the loss of X-ray dose by holder absorption^17^. As the sample holder size was small and magnetic stirring was not possible. So, the 6D sample holder stage has been utilized to uniformly scan the full area by continuous constant 2D motion^33,34^ as shown in Fig. 4a. This technique produces the similar effect as of stirring during the reaction.

At first, the reaction was carried out without any scavenging agent and extinction spectra of prepared samples has been investigated for their respective plasmonic effects as a function of X-ray irradiation time as shown in Fig. 4b,c. The change in the color (for Au: greenish-yellow to pink, and for Ag: transparent to yellow) during the irradiation is attributed to their respective *LSPR*-response and the first visible optical confirmation for the formation of plasmonic nanostructures^43^. The relative variation in the color of samples as a function of irradiation time is shown inset of Fig. 4b,c. The extinction spectra of both *Au* and *Ag*-salts irradiated with X-rays show characteristics plasmon band at $\sim 550$ nm and $\sim 420$ nm respectively^44^, which confirms the formation of nanostructures at 6.7  keV X-ray irradiation. The maxima in the extinction spectra is attributed to the transverse oscillation of electrons due to the absorption as well as scattering of light at particle surface, whereas minima at $\sim 500$ nm in *Au* and $\sim 330$ nm in *Ag*-case to the imaginary part of size dependent refractive index $(n_{Im}=Im([\epsilon (\lambda )]^{1/2})$^45^. The intensity of the plasmon-band increases with the irradiation time clearly depicting the rise in concentration of nanoparticles with time^42,44,46^ as shown in Fig. 4d,e. The time took to complete the reaction is depend on the sample holder size, so it is been recommended to select an appropriate design for radiation induced synthesis. The sample holder size can be made comparable to the beamsize and precursor solution must be allowed to flow at constant rate for better results. The plasmon-band position (centroid) and broadening (FWHM) in extinction spectra of $M-NP$ belongs to particle size, shape, and surface charge^47^. In case of the Au-nanostructures, a shift in the plasmon-band towards higher wavelength is observed, whereas edge shift to lower wavelength following the similar trend as in *Ag*-nanostructures. The trend of shift in plasmon-frequency is attributed to the size growth alongwith the production of smaller size particle as function of irradiation time^46^, which is in agreement with the theory of radiolysis based nanostructures synthesis.

**Figure 5:**
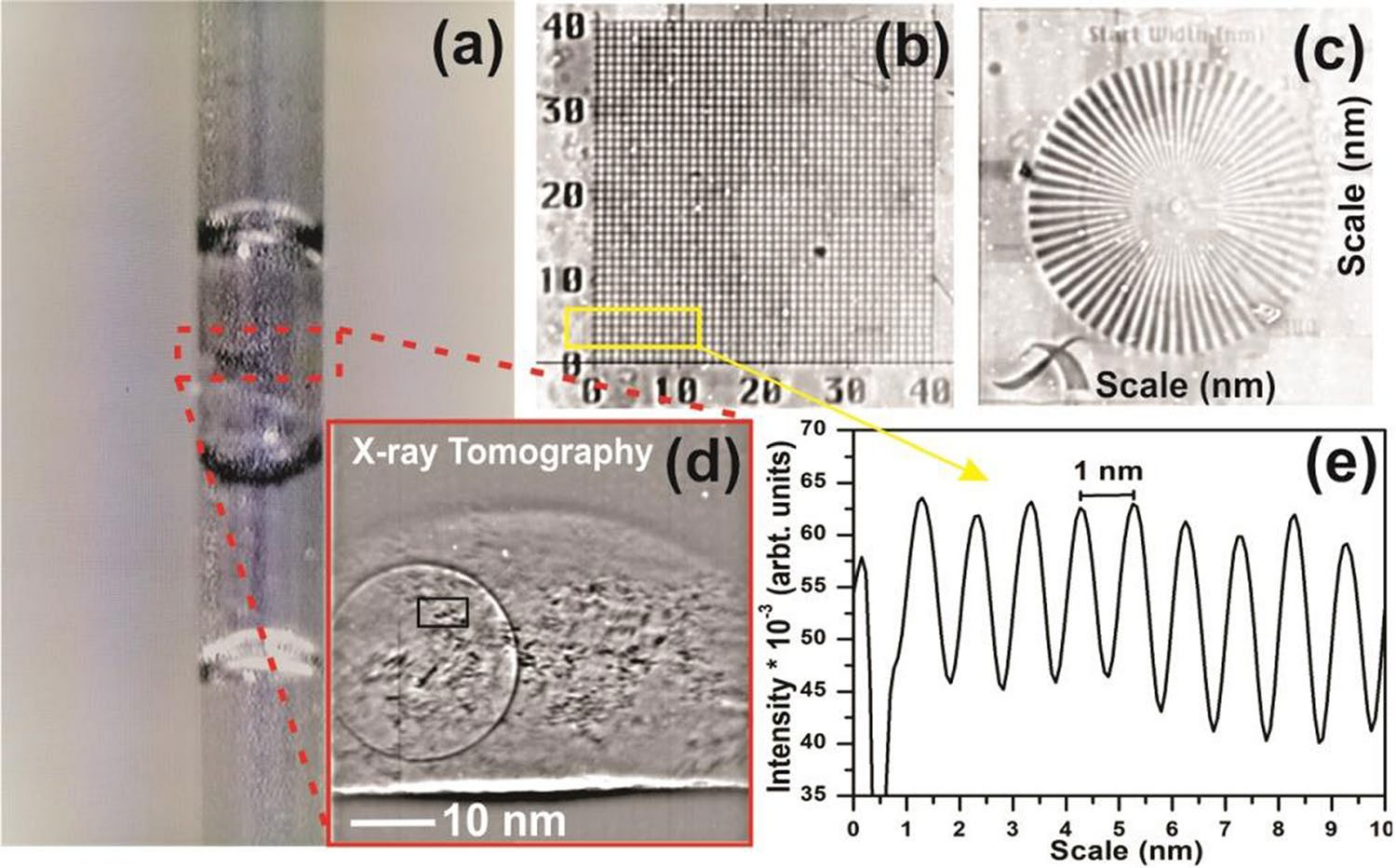
Nanotomography of plasmonic nanostructures: optical in-situ imaging of Kapton capillary filled with metal-salt precursor solution during the X-ray irradiation (**a**), X-ray imaging of scale pattern (**b**,**c**), in-situ X-ray imaging of Au-nanostructures (**d**), sectional line-scale pattern (**e**), respectively.

Simultaneously, X-ray tomography of the irradiated sample has been recorded as shown in the Fig. 5. The monochromatic X-ray beam has been focused on a point where the high flux of formed nanoparticles was observed by in-situ optical imaging. In the scintillator based detection system, three modes of beams were detected i.e., positive first-order, zeroth-order, and negative first-order image. Since the resolution is high enough, so the arrangement is able to separate them and collect only the positive first-order image beam. The kapton-polyimide tube is placed in the vertical position and the formed particles are highly mobile as observed by optical imaging, might be because of the effective zeta-potential at the nanoparticle surface^42^. The X-ray imaging of the reference-scale target depict the resolution of beamline and detection system as shown in Fig. 5. The image-date was calibrated using reference-target to performed particle-size and there distribution analysis. The detector system is allowed to capture the diffracted X-rays for 1 s after 5 min of exposure/irradiation. One offsite image has been captured which was subtracted from the final image to remove background effects. The obtained X-ray images were processed by the $ImageJ-1.41o$ software^48^.

**Figure 6:**
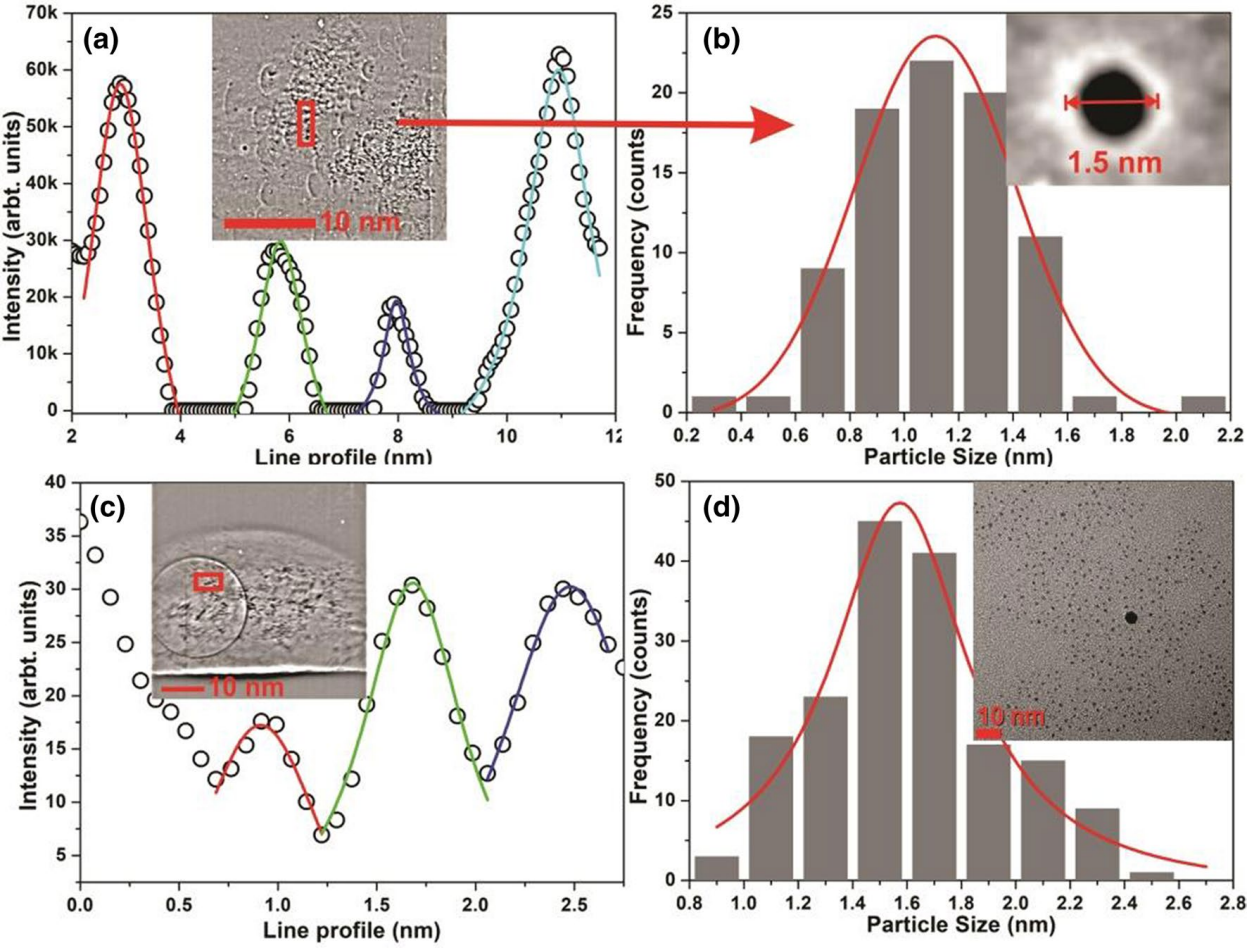
Real-time nanotomography of Plasmonic nanostructures (**a**–**c**) along-with sectional line-profile fitting (**a**,**c**) and size-distribution histogram (**b**), inset shows the single particle captured by X-ray imaging. TEM image and size-distribution of sample for comparison (**d**).

The images were treated with the bandpass filters, threshold, brightness, and contrast filters. Figure 5b,c shows the X-ray image of the reference scale target and the corresponding sectional line-profile showing 1 nm spacing in the grid-graph reveals the high resolution of system geometry. Figure 6a–c. shows the final real-time obtained X-ray image of *Au*-nanoparticles. The circular rings in the X-ray images (Fig. 6a,c) are observed because of the scattering by the air-bubbles produced during the irradiation as also detected by in-situ optical-imaging. The sectional line profile of the X-ray images shows the particle size of $\sim$ 1–2 nm, and the corresponding size-distribution histogram reveals the average particle-size of $1.12\pm 0.29$ nm with $26.58\%$ polydispersity. The inset (Fig. 6b) shows the single *Au*-nanoparticle captured by X-ray imaging confirms the spherical nature of particles. The same sample has also been investigated by transmission electron microscopy (TEM), and the results shows the spherical *Au*-nanoparticles of averaged particle size $1.57\pm 0.30$ with $19.34\%$ polydispersity as shown in Fig. 6, which is in good agreement with X-ray tomography.

As per the literature, it’s the first time real-time/in-situ X-ray nanotomography of the plasmonic nanostructures has been successfully achieved using a single monochromatic synchrotron X-rays beamline. With the growing electronics and continuously improving instrumentation, one could precisely control the size and quality of plasmonic nanoparticles using better X-ray imaging resolving power. Therefore, for bio-applications it would be great idea to synthesise plasmonic nanoparticles at active-site to get enhanced activity to target tissues without harming the other beneficial bacterias^49,50^.

**Figure 7:**
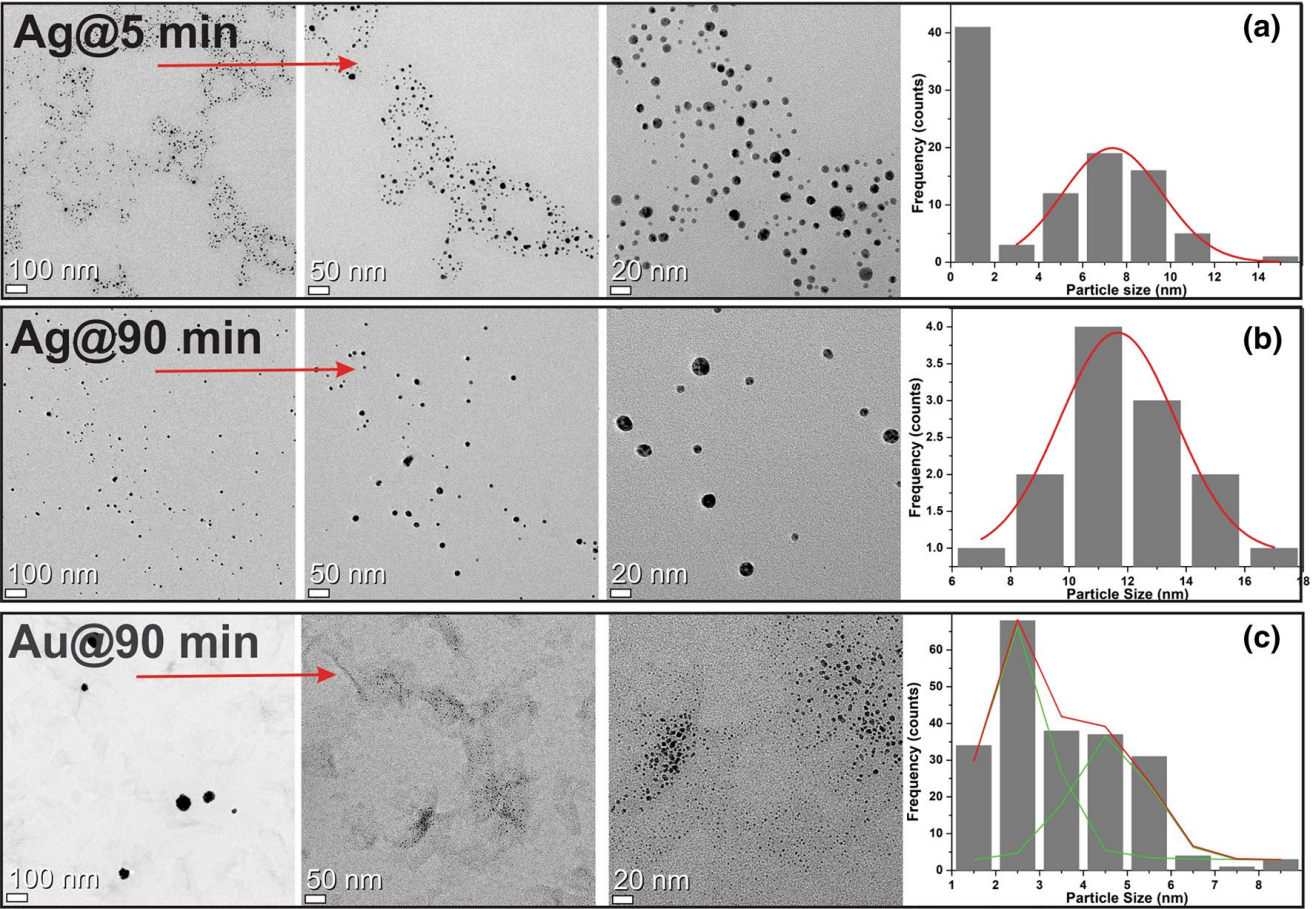
Transmission electron microscope (TEM) images of Ag and Au-nanostructures obtained after the irradiation of 5 min (**b**) and 90 min irradiation (**c**) at different resolution scale.

The lowest and highest dose irradiated samples of *Au* and *Ag* have been studied using TEM as shown in Fig. 7. It has been observed that there were two generation of particles. The small-size particles in the vicinity of radiation path and coalesce to grow particle size as moves from the radiation track under the effect of its own zeta-potential. Therefore the average particle-size grows from $7.35\pm 2.25$ nm to $11.65\pm 2.32$ nm for *Ag* and $1.57\pm 0.30$ nm to $4.61\pm 0.13$ nm for *Au* and polydispersity decreases from $30.59\%$ to $19.95\%$ for *Ag* and $19.34\%$ to $5.32\%$ for *Au* with the increase in integrated-dose from $3.21-77.18 \,KGy$. It is confirmed from the results that the reduction starts within a minute of X-ray irradiation, which would make this technique dominate over the conventional methods.

**Figure 8:**
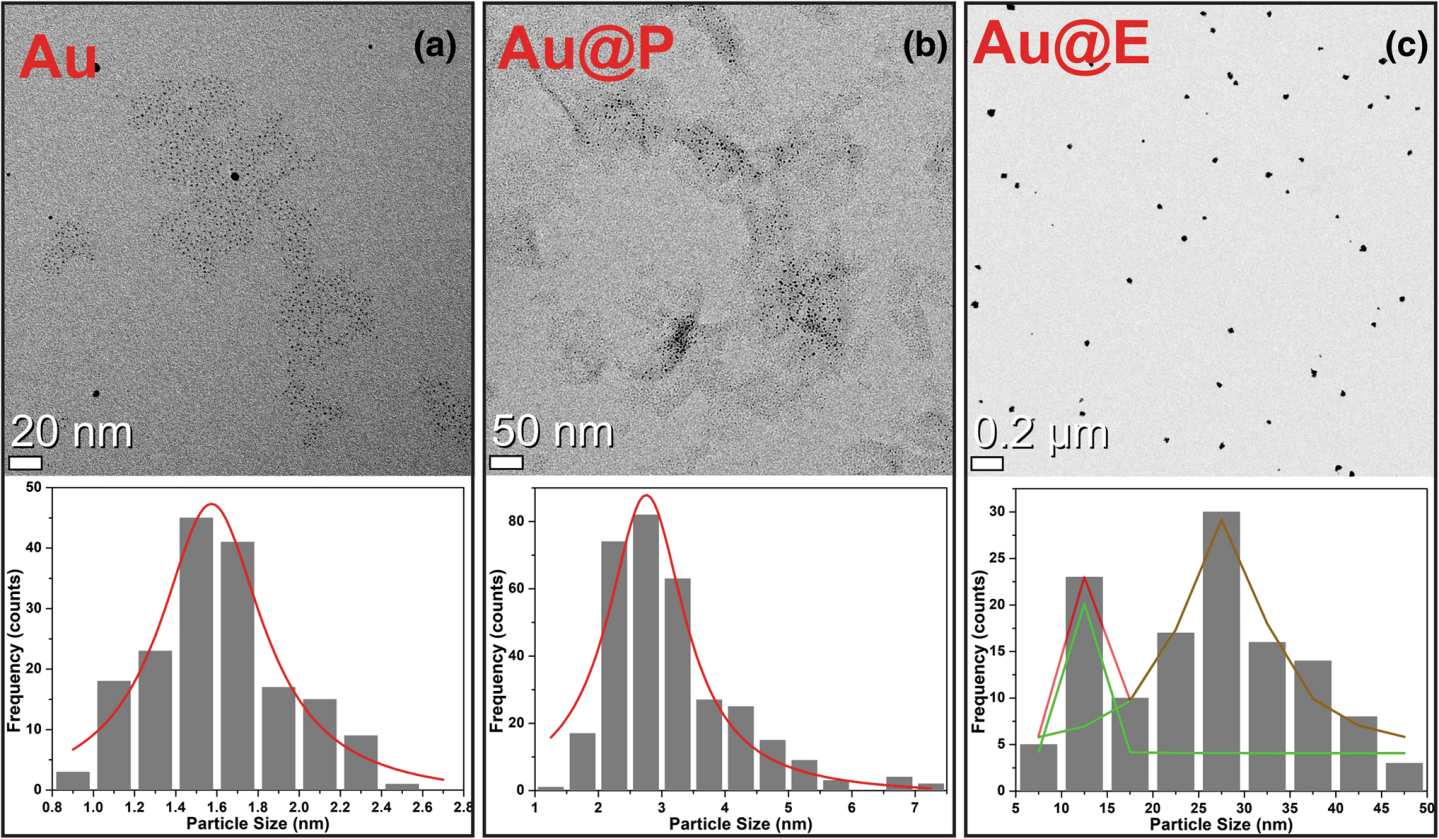
Transmission electron microscope (TEM) images of Au-nanostructures and respective size-distribution, synthesized under different conditions.

Furthermore, the effect of surrounding media on the particle-size evolution has been investigated. Three precursor samples of $AuCl_3$(a), $AuCl_3+PVP$(b) and $AuCl_3+Ethanol$ in deionized-water were irradiated at 6.7  keV X-rays at constant integrated dose. The average particle size of *Au*-nanoparticles is $1.56\pm 0.30$ nm for (a), $2.75\pm 0.74$ nm for (b) and $27.63\pm 0.29$ nm for (c) clearly reveals that the surrounding media significantly affect the particle-size as shown in Fig. 8. In case of sample (a) there might be a chance of oxidation (reverse reaction) under $OH^\bullet$ radicals. Whereas *PVP* has enough ability for surface capping and stabilize the nanoparticles results to prevent the oxidation^16,17^. Moreover the addition of Ethanol $(CH_3CH_2OH)$ not only scavenge $OH^\bullet$ radical but also simultaneously produces the $CH_3C^\bullet$ radical^42^ which act as strong reducing agent, therefore the reduction rate increases in sample (c) resulting in particle size growth.

## Conclusion

In this work, real-time/in-situ synthesis and detection of plasmonic metal (Au and Ag) nanoparticles have been successfully achieved by Synchrotron monochromatic 6.7  keV X-rays based Nano-Tomography technique. The experiments were performed during the irradiation process using a single X-ray beamline. With the successful outcome of real-time X-ray nano-tomography of plasmonic nanostructures at such a low energy (6.7  keV) would lead to the possibility of these type of experiments at laboratory-based tabletop sources. The X-ray irradiation induced radiolysis of water molecule resulting in the production of $e_{aq}^-,\,OH^\bullet ,$ and $O_2^-$ was confirmed by in-situ high resolution optical imaging detectors. The results clearly revealed the production of mono-disperse plasmonic nanoparticles and the particle size can be well-controlled by X-ray dose and surrounding media. With our best knowledge, this is the first experiment utilizing the single X-ray beam for reduction of $M^+$ to $M^0$ under water radiolysis and in-situ/real-time imaging characterization. This work provided the protocol for real-time detection of nanostructures and precisely control the size of nanostructures under any kind radiation ($\mathrm{X-rays}, \,\gamma \mathrm{-rays}, \,\mu \mathrm{-wave}$,  electron/proton  etc.) or chemicals induced reduction of metallic-salts to synthesize the high-purity grade monodisperse nanoparticles that would enhance the plasmonic photovoltaic application. This work also led to the phase-contrast imaging of active-site cancer tissue without harming other body-tissues.
